# Can Vascular Endothelial Growth Factor and Microvessel Density Be Used as Prognostic Biomarkers for Colorectal Cancer? A Systematic Review and Meta-Analysis

**DOI:** 10.1155/2014/102736

**Published:** 2014-03-27

**Authors:** Yibaina Wang, Xiaoping Yao, Jie Ge, Fulan Hu, Yashuang Zhao

**Affiliations:** Department of Epidemiology, Public Health College, Harbin Medical University, 157 Baojian Street, Nangang District, Harbin, Heilongjiang 150081, China

## Abstract

*Background.* Vascular endothelial growth factor (VEGF) and microvessel density (MVD) are associated with greater incidence of metastases and decreased survival. Whether they can be used as prognostic indicators of colorectal cancer (CRC) is still controversial. *Methods.* The authors performed a meta-analysis using the results of a literature search of databases of PubMed and EMBASE, and the references of articles included in the analysis. Meta-analysis was performed using random effects model and hazard ratios (HRs) and 95% confidence intervals (CIs) as effect measures. *Results*. Twenty studies contributed to the analysis of VEGF, of which 16 were used for overall survival (OS) and 9 for disease-free survival (DFS). High VEGF levels has a relationship with unfavorable survival (OS: HR = 1.98, 95% CI: 1.30–3.02; DFS: HR = 2.10, 95% CI: 1.26–3.49) and a 4.22-fold increase in the rate of distant metastases. Analysis was performed on 18 studies for MVD; the results showed that patients with high MVD expression in tumors appeared to have poorer overall survival (HR = 1.39, 95% CI: 1.22–1.58) and were at a greater risk of having unfavorable clinical characteristics related to prognosis. Corresponding results were obtained from quantitative and/or qualitative analysis of clinicopathological. *Conclusions*. The meta-analysis demonstrates that VEGF and MVD can be used as prognostic biomarkers for CRC patients.

## 1. Introduction

Neoangiogenesis, the formation of new blood vessels from existing ones, plays an important role in the growth and progression of tumors [1]. As a tumor grows, the lack of a sufficient blood supply creates a hypoxic environment that stimulates the release of factors such as hypoxia inducible factor- (HIF-)1*α* to induce angiogenesis by activating the transcription of vascular endothelial growth factor (VEGF) [2, 3]. VEGF is an endothelial, cell-specific mitogen and an angiogenic inducer, as well as a mediator of vascular permeability that was first recognized by Leung et al. in 1989 [4]. Along with fibroblast growth factors, transforming growth factors, tumor necrosis factor, interleukin-8, and various angiopoietins, VEGFs are potent inducers of the angiogenic switch. This switch is characterized by a sequence of steps beginning with vessel dilation and the detachment of pericytes from preexisting vessels followed by angiogenic sprouting and the proliferation of endothelial cells, new vessel formation, and recruitment of perivascular cells [5]. The use of microvessel density (MVD) as a surrogate marker for tumor angiogenesis has also been reported.

Bevacizumab is a specific anti-VEGF drug that has recently attracted the attention of clinical oncologists. A series of multicenter, randomized controlled clinical trials have evaluated the efficacy and safety of bevacizumab when used in the survival of patients with metastatic colorectal cancer (CRC) [6–9].

A meta-analysis including 10 VEGF studies and 18 MVD studies was conducted by be G. Des Guetz 2006; this meta-analysis suggested that the expression of both VEGF and MVD significantly associated with poor overall survival (OS) for colorectal cancer [10]. However, the study measured the effect by using relative risk, which only measures the number of events and does not illustrate the time they occur. Hazard ratios (HRs) take into account the number and timing of events, but the time until last follow-up for each patient who has not experienced an event is censored [11]. Since 2006, several additional studies using larger sample sizes and multivariate Cox analyses have been reported. The authors conducted a systematic review of these new data by performing a meta-analysis, which for the first time used pooled HR data to evaluate whether VEGF or MVD expression was associated with prognosis in CRC.

## 2. Materials and Methods

### 2.1. Search Strategy and Selection Criteria

A comprehensive, systematic literature review was performed independently by Y.B.N.W. and X.P.Y. from the inception to February 30, 2013, using PubMed, EMBASE, and the Cochrane database to identify potentially relevant studies. The structured search was based on four main medical subject headings and keywords: microvessel density, VEGF, colorectal cancer, and prognosis.

Based on careful reading of the abstracts, studies were selected for further analysis. The authors manually reviewed all of the reference lists for the selected studies to identify additional articles for inclusion. This cross-referencing strategy was performed until no further eligible publications were identified.

To be included, the studies had to meet the following criteria: the original study had to be designed to assess the association between the expression of VEGF or MVD and the prognosis of CRC patients, and the study had to provide primary outcomes on survival, such as HRs for Cox proportional hazard model. Only English language studies were included.

To avoid overlapping data in duplicate publications, we analyzed all of the author names for each study, the different medical institutions involved, and the timeframe of the research. In the case where multiple studies using the same patient population were identified, the combined data were included. We then adapted a validity questionnaire to weight the quality and applicability of the studies included (see Online Resource 1 in the Supplementary Material available online at http://dx.doi.org/10.1155/2014/102736) [12].

### 2.2. Data Extraction

Two reviewers independently extracted the relevant data from the full texts of all included studies according to a predefined protocol. The standardized data-extraction form consisted of the following items: first author, year of publication, characteristics of the study population, duration of follow-up, counting method for VEGF and MVD, cut-off values for VEGF and MVD, HRs and their 95% confidence intervals (CIs) on OS, and/or disease-free survival (DFS) between the high and low groups in multivariate analysis. Differences in extracted data were crosschecked until consensus was reached.

### 2.3. Statistical Analysis

Pooled HRs and their 95% CIs on OS and/or DFS between the high and low groups were calculated using the maximum adjusted HR for each included study. To estimate the effect of VEGF or MVD on the prognosis of the CRC patients, the parameters were considered or transformed as binary variables using the cut-off value for the parameter (usually the median) [13]. The heterogeneity among studies in the meta-analysis was evaluated by *I* ^2^ statistics and found to be *I* ^2^ > 50%; as this was considered to be unacceptable, a random effects model was adopted [14]. To assess the influence of publication year, the authors conducted a cumulative meta-analysis in which they accumulated the included studies chronologically by year of publication. Sensitivity analysis was performed by comparing the combined results before and after one study was sequentially removed from the meta-analysis. Subgroup analyses were conducted by age, geographic area, and duration of follow-up to evaluate all conceivable sources of heterogeneity. The authors also evaluated the pooled association between VEGF and MVD expression and different clinical characteristics, both qualitatively and quantitatively. Additionally, the papers used funnel plots to detect publication bias and further qualitative bias using Egger's regression test and Begg's rank correlation test [15]. If bias was present, Duval and Tweedie's trim-and-fill method was adopted to adjust the original pooled result. All statistical analyses were performed using Comprehensive Meta-Analysis version 2.0.

## 3. Results and Discussion

### 3.1. Literature Search and Description of Included Studies

**Figure 1:**
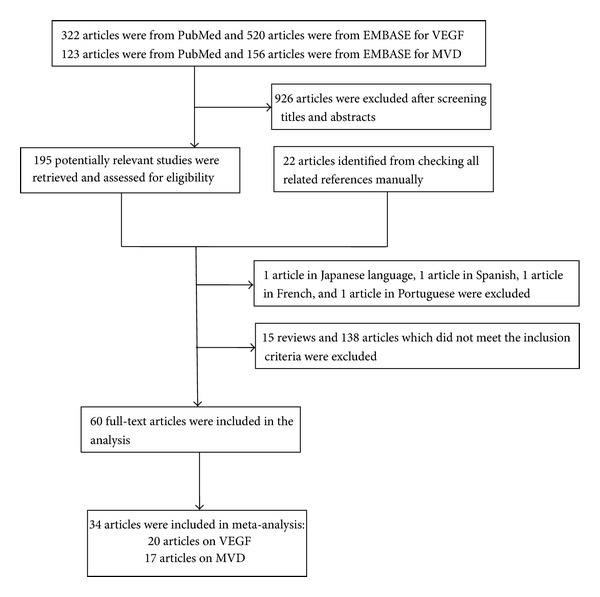
The flow diagram of the studies search.

Figure 1 shows the flow diagram for the selection of included studies. The systematic literatures search yielded 1,143 total articles, 1,083 of which were excluded either because of a lack of survival data or an overlap with other studies. Sixty references were used in the analysis of association between VEGF/MVD expression and clinical and pathology feature; 34 articles met the inclusion criteria and were included in the final meta-analysis. Online Resources 2 and 3 summarize the baseline characteristics and quality scoring of these studies.

The final 34 studies represented 3,618 patients and ranged in sample size from 31 to 278 (median, 101 patients). Three studies [1, 16, 17] included primary CRC only, 6 studies [18–23] focused only on advanced patients, and the remaining studies examined patients at all stages of disease.

VEGF and MVD expression were assessed in CRC specimens excised before treatment with either chemotherapy or preoperative radiotherapy, except in four studies [18, 19, 22, 24].

The methodological quality scores reflect the high quality of the included studies; only 7 studies were determined to be level c, meaning they were imperfect in the research design, lab methodology, or statistical analysis.

### 3.2. VEGF Expression and Prognosis for CRC

**Figure 2:**
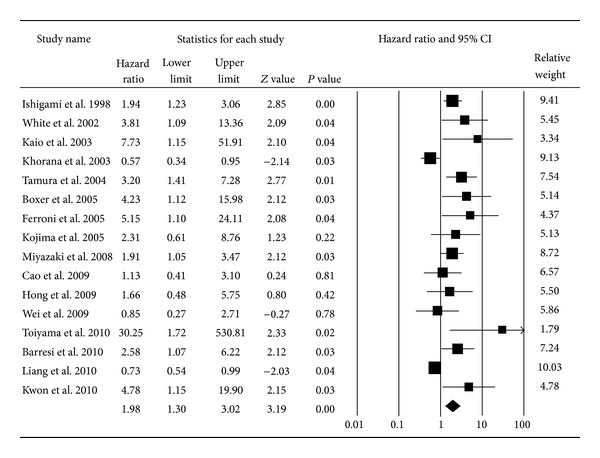
Forest plot for the association between VEGF expression and overall survival of CRC. The squares and horizontal lines represent the HRs and 95% confidence intervals (CIs) in the individual studies; the size of each square represents the weighting assigned to the corresponding study. The diamond indicates the pooled HRs.

**Figure 3:**
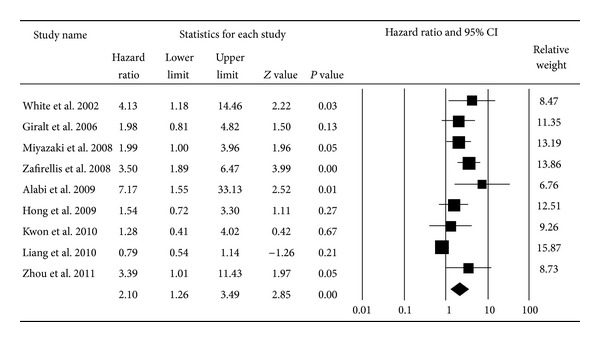
Forest plot for the association between VEGF expression and disease-free survival of CRC.

**Figure 4:**
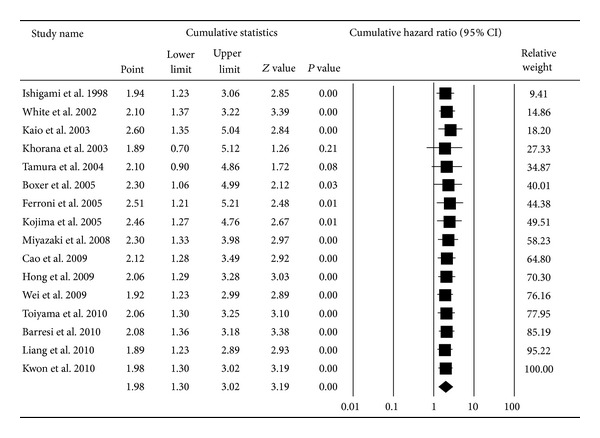
Cumulative meta-analysis for the association between VEGF expression and overall survival of CRC.

**Figure 5:**
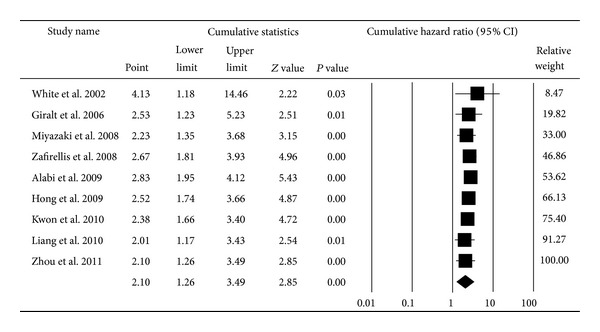
Cumulative meta-analysis for the association between VEGF expression and disease-free survival of CRC.

Based on the study data available for evaluating the association between VEGF expression and prognosis, the pooled HR was 1.98 (95% CI: 1.30–3.02; *P* < 0.01; *I* ^2^ = 73.64) for overall survival [1, 16, 18, 24–36] and 2.10 (95% CI: 1.26–3.49; *P* < 0.01; *I* ^2^ = 71.44) for disease-free survival [18, 19, 26, 27, 31, 35, 37–39] based on the random effects model (Figures 2 and 3). The cumulative meta-analysis showed that the strength of this association varied by approximately 2.00. However, the variant trended small, and the pooled result approached stability with time (Figures 4 and 5). Further subgroup analyses were carried out based on age at diagnosis, nationality, and the duration of follow-up. VEGF expression has a relationship with OS both in the elderly group (pooled HR = 2.18, 95% CI: 1.11–4.30; *P* = 0.02) and the nonelderly group (pooled HR = 1.91, 95% CI: 1.05–3.46; *P* = 0.03) (*P* = 0.77 for comparison). The prognostic effect was more apparent in those of European ancestry (pooled HR = 3.42, 95% CI: 1.90–6.14; *P* < 0.01) than among Asians and Americans (*P* < 0.01). Additionally, the association between VEGF and survival was stronger in the four studies with a median follow-up time longer than 60 months (pooled HR = 3.90, 95% CI: 2.15–7.07; *P* < 0.01) than in studies with a follow-up period shorter than 60 months (pooled HR = 1.39, 95% CI: 0.81–2.36; *P* = 0.23) (*P* = 0.01 for comparison) (Table 1).

The associations between VEGF expression and clinical features were provided by 19 studies [1, 19, 29–33, 40–51] (Table 2). Notably, higher rates of VEGF expression were significantly associated with the following clinicopathological features: lymph node metastasis (pooled OR = 2.51, 95% CI: 1.51–4.15; *P* < 0.01) and vascular metastasis (pooled OR = 2.38, 95% CI: 1.49–3.79; *P* < 0.01). Similarly, the incidence of tumor distant metastases tended to be higher in patients with high rather than low expression of VEGF (pooled OR = 4.22, 95% CI: 2.93–6.06; *P* < 0.01).

### 3.3. MVD Expression and Prognosis for CRC

**Figure 6:**
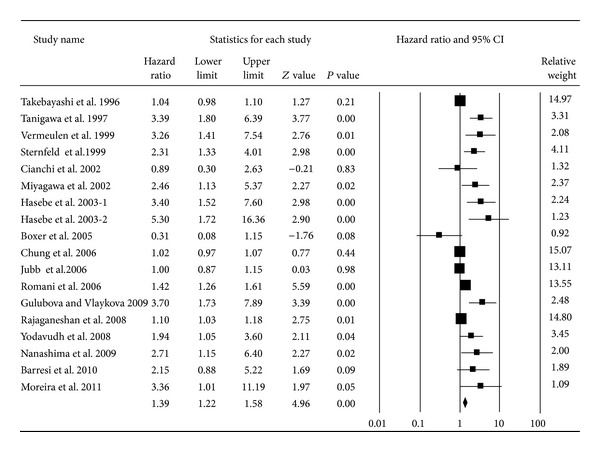
Forest plot for the association between MVD expression and overall survival of CRC.

**Figure 7:**
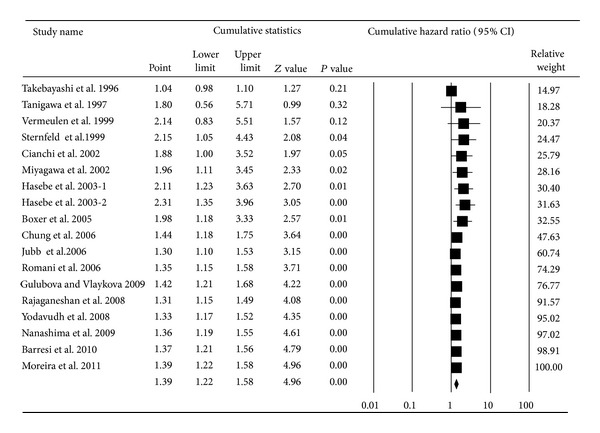
Cumulative meta-analysis for the association between MVD expression and overall survival of CRC.

The MVD meta-analysis using 18 studies [1, 3, 16, 17, 21–23, 27, 52–60] showed that MVD was associated with overall survival with a pooled HR of 1.39 (95% CI: 1.22–1.58; *P* < 0.01; *I* ^2^ = 83.14) (Figure 6). Additionally, the cumulative meta-analysis demonstrated a decreasing association over time that eventually tended to be statistically significant and stable (Figure 7). Subgroup analysis found that the significant associations remained in the nonelderly patients (pooled HR = 2.17, 95% CI: 1.23–3.83; *P* < 0.01) but not in the elderly patients (pooled HR = 1.28, 95% CI: 0.94–1.74; *P* = 0.11). However, the difference between the two groups was not statistically significant (*P* = 0.11 for comparison). The pooled HR in Asians (pooled HR = 1.35, 95% CI: 1.12–1.63; *P* < 0.01) was similar to that of Europeans (pooled HR = 1.62, 95% CI: 1.24–2.12; *P* < 0.01). Only two American reports studied the association between MVD and the prognosis of CRC, yielding a pooled HR of 1.57 (95% CI: 0.50–4.95; *P* = 0.44) (*P* = 0.55 for comparison). The authors also noted that different durations of follow-up did not directly affect the association between MVD and survival; the pooled HR for follow-up longer than 60 months (pooled HR = 1.60, 95% CI: 1.19–2.16; *P* < 0.01) was similar to the value for follow-up of less than 60 months (pooled HR = 1.35, 95% CI: 1.14–1.60; *P* < 0.01) (*P* = 0.33 for comparison) (Table 1).

Further examination of the associations between MVD and clinicopathological factors used 25 studies [1, 3, 16, 17, 23, 32, 46, 49, 54, 55, 57, 61–74]. The analysis revealed a more pronounced blood vessel density value in patients with vascular metastasis compared to those without (pooled OR = 1.43, 95% CI: 1.06–1.92; *P* = 0.02; mean difference (MD): 13.99; standardized mean difference (SMD): 0.60) and in patients with lymph node metastasis compared to those without (pooled OR = 1.84, 95% CI: 1.19–2.85; *P* < 0.01; MD: 7.97; SMD: 0.45). Similarly, an increased incidence of distant metastases was seen in those with a high level of MVD expression compared to those with low expression (MD: 13.14; 1.43) (Table 3).

### 3.4. Sensitivity Analysis

Based on studies of the association between VEGF and prognosis, each article had a balanced weight of less than 10%. However, in 18 studies on MVD, 3 studies provided the largest weight in the analysis; further sensitivity analysis after removing these studies by Rajaganeshan et al. [22], Chung et al. [21], and Takebayashi et al. [59] did not substantially impact the pooled results. When the analysis was restricted to studies where specimens were collected before chemotherapy, the pooled results for VEGF and MVD did not substantially change (pooled OR_OS-VEGF_ = 1.89, 95% CI: 1.23–2.92; *P* < 0.01; pooled OR_DFS-VEGF_ = 2.13, 95% CI: 1.21–3.77; *P* < 0.01; pooled OR_OS-MVD_ = 1.53, 95% CI: 1.30–1.79; *P* < 0.01).

### 3.5. Publication Bias

**Figure 8:**
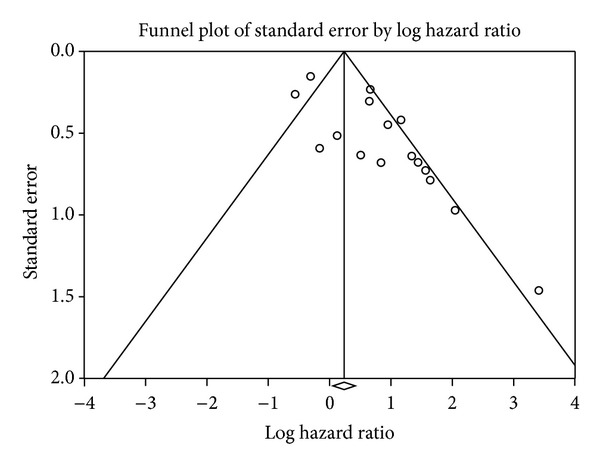
Publication bias pooled association between VEGF expression and overall survival.

The presence of publication bias for the pooled association between VEGF expression and the prognosis of CRC was demonstrated visually using a funnel plot (Figure 8) and further qualitative analyses using Begg's rank correlation test or Egger's regression test. Additional analysis using Duval and Tweedie's trim-and-fill method found that the adjusted summary HRs did not change the results (HR = 1.68, 95% CI: 1.12–2.52).

**Figure 9:**
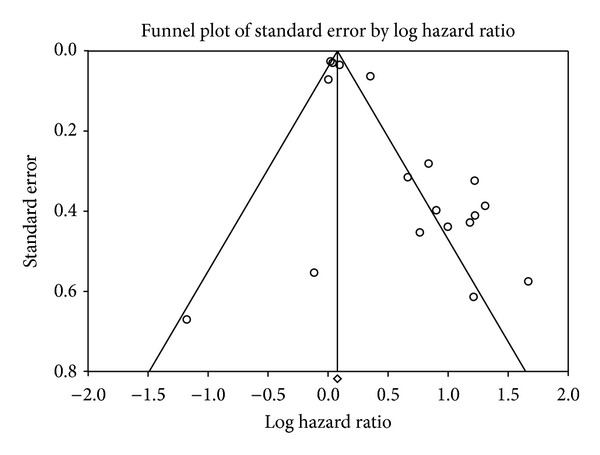
Publication bias pooled association between MVD expression and overall survival.

The degree of asymmetry for the funnel plot (Figure 9) of the individual study results around the combined HR for OS between MVD expression and prognosis of CRC suggested that there was some degree of publication bias; this finding was confirmed by Egger's regression test (*P* < 0.05). Additional analysis was performed using Duval and Tweedie's trim-and-fill method, and it was found that the summary HRs (HR = 1.27, 95% CI: 1.11–1.46) did not change the result, although the estimate of effect size tended to be small after adjustment.

## 4. Discussion

As the predominant angiogenesis factors in the growth and maturation of new vessels, VEGFs are associated with greater incidence of metastases and decreased survival. Results from previous studies assessing the relationship between VEGF, MVD, and the survival of CRC have been inconsistent. Until now, however, no meta-analysis has been conducted to evaluate the prognostic effects of VEGF/MVD using HR as an indicator, although a meta-analysis using OS and DFS was published by Des Guetz et al. 2006 [10]. It is known that HR is a more powerful factor than OS and DFS in survival data analysis [11]. The current systematic review and meta-analysis was therefore performed to determine whether the expression levels of VEGF and MVD in biopsy samples could accurately predict the prognosis of CRC patients. Our analysis, which pooled the maximum adjusted HRs from proportional hazard regression models, provided clear evidence that the expressions of VEGF and MVD were unfavorable prognostic predictors in colorectal cancer. Notably, overexpression of VEGF correlated with nearly two times the risk of death. Similarly, overexpression of MVD increased the risk of death in CRC patients by 39%. Unfortunately, sufficient data on MVD and disease relapse could not be retrieved from existing studies. Because multivariate adjusted HRs avoid, as much as possible, confounding factors such as clinicopathological features, the authors concluded that both VEGF and MVD can independently provide valuable prognostic information for CRC patients, although the effect of MVD was weaker than VEGF.

Our data were consistent with the results of previous meta-analyses. The results from one meta-analysis of 51 studies demonstrated that the overexpression of VEGF was significantly correlated with poor prognosis for patients with lung cancer [75]. Smith et al. [76] demonstrated that the expression of VEGF represented a significant and reproducible marker for adverse prognosis in resected pancreatic cancer as well. Similar results were also found in patients with hepatocellular cancer [77], breast cancer [13], and gastric cancer [78].

The papers further noticed that VEGF had a stronger association with overall survival of CRC in Europeans than in Asians. Our meta-analysis is the first to report this phenomenon. Further studies are needed to prove that the phenomenon exists and explore whether the difference is due to genetic susceptibility or other factors. When using age as a grouping factor, the effects of VEGF and MVD on prognosis are inconsistent, and the reasons for this remain unclear. The authors also observed that the duration of the follow-up period had an effect on the ability of VEGF and MVD to predict prognosis for CRC. The effect of VEGF or MVD as a significant prognostic indicator was much more obvious in those studies where the median follow-up period was longer than 60 months than in those with median follow-up periods shorter than 60 months.

In CRC patients, important prognostic parameters such as the TNM staging system, tumor grade, vascular-lymphatic invasion, and lymph node involvement have been identified. Thus, to understand the influence of VEGF and MVD expression on prognosis for CRC, we also assessed their relationship to clinicopathological variables. We observed that elevated VEGF apparently associated with more severe vascular-lymphatic invasion and positive lymph node metastasis. Moreover, patients with high VEGF levels had a 4.22-fold greater risk of developing distant metastases compared to patients with lower VEGF levels. Additionally, the analysis revealed that patients with increased MVD levels correlated with greater tumor aggressiveness, including poor differentiation and higher frequencies of vascular-lymphatic invasion, lymph node invasion, and distant metastases. The association between tumor angiogenesis and clinicopathologic factors thus provides further evidence that using VEGF and MVD as indicators for the prognosis of CRC is feasible.

Malignant tumors are crucially dependent on the process of angiogenesis. The key promoter of angiogenesis is VEGF, which may correlate with advanced clinical stage and worse prognosis [79]. The novel antiangiogenic agent, bevacizumab, is a recombinant, humanized monoclonal antibody that specifically blocks the activity of VEGF [80]. Large, randomized placebocontrolled trials [8, 9, 81, 82] and two pooled analyses [83, 84] have demonstrated that the addition of bevacizumab to traditional chemotherapy regimens provides statistically significant and clinically meaningful improvements in overall survival as well as response rate. However, little information about the relationship between VEGF status and survival outcomes in patients with CRC treated with bevacizumab was provided. Future studies will be needed to validate whether VEGF can be used as a biomarker to select patients who may benefit from bevacizumab and whether dose modification of bevacizumab may vary according to the degree of VEGF expression.

## 5. Limitations

Several limitations of this meta-analysis should be considered. First, the results should be interpreted in the context of treatment, but treatment was not uniform. Nonetheless, it is unlikely that the use of chemotherapy differed substantially based on the expression of VEGF or MVD. Thus, we concluded that the confounding effects of therapy would not have been substantial.

The next limitation was between-study heterogeneity. The data after subgroup analysis suggest that heterogeneity may be partly attributable to the grouped variable even though heterogeneity still existed. We believe that the observed discrepancy might be accounted for by the following: (1) the diversity across studies in patient selection, even though primary patients accounted for 47% of the total number in all studies; (2) the nonstandardized methodologies for assessing VEGF or MVD expression, such as the counting methods and whether or not assessors were blinded to the clinical data; and (3) the absence of uniform cut-off criteria for the studies  (Online Resource  4);  However, using the random effects model helped to diminish the effect of observed heterogeneity and led to results that were more conservative.

At last the meta-analysis was subject to publication bias; we attempted to overcome this limitation by identifying all of the relevant studies in key publications and adopting rigid inclusion criteria.

## 6. Conclusion

In conclusion, our systematic review and meta-analysis provided persuasive evidence that VEGF and MVD could be used as prognostic biomarkers for colorectal cancer. High expression levels of VEGF and MVD yielded poor prognosis results for patients after CRC surgery and indicated adverse effects on oncological clinical outcomes as demonstrated by the increased rates of vascular-lymphatic and lymph node metastasis and the increased incidence of distant metastases. The results can guide postoperative treatment of patients, especially the application of the bevacizumab.

## Supplementary Material

Online Resource 1 showed the quality assessment questionnaire, which was used to weight the quality and applicability of the included studies from research design, laboratory methodology and analysis of the results.
Online Resources 2 summarized the main characteristics of the VEGF related studies, and the characteristics included detail information of the study population, median months of follow-up, HR value, adjusted variable in Cox analysis and quality scoring of each article.
Online Resources 3 summarized the main characteristics of the MVD related studies, and the characteristics included detail information of the study population, median months of follow-up, HR value, adjusted variable in Cox analysis and quality scoring of each article.
Online Resources 4 described the determination method of cut-off criteria and the specific cut-off value for VEGF/MVD in each study. And the cut-off value was used to assess the expression level of VEGF or MVD.

## Figures and Tables

**Table 1:** Pooled HR and subgroup analysis on the association between VEGF/MVD and survival of CRC comparing the high group with the low group and publication bias.

Subgroups	Number of studies	Pooled HR (95% CI)	*P* value	*I* ^2^	Publication bias				Between subgroup *P* value
					*P* for Begger	*P* for Egger	Number of trim and fill	Adjusted HR (95% CI)	
VEGF (OS)	16	1.98 (1.30–3.02)	<0.01	73.64	0.02	<0.01	5 left	1.51 (1.02–2.23)	
Racial descent		1.46 (1.08–1.98)	0.01	73.64	—	—	—	—	
America	1	0.57 (0.34–0.95)	0.03	0.00	—	—	—	—	<0.01
Asia	11	1.90 (1.17–3.09)	0.01	72.09	—	—	—	—	
Europe	4	3.42 (1.90–6.14)	<0.01	0.00	—	—	—	—	
Age		2.02 (1.29–3.16)	<0.01	73.64	—	—	—	—	
<65	9	1.91 (1.05–3.46)	0.03	73.71	—	—	—	—	0.77
≥65	7	2.18 (1.11–4.30)	0.02	75.01	—	—	—	—	
Follow-up		2.19 (1.47–3.26)	<0.01	77.97	—	—	—	—	
<60 months	8	1.39 (0.81–2.36)	0.23	78.14	—	—	—	—	0.01
≥60 months	4	3.90 (2.15–7.07)	<0.01	0.00	—	—	—	—	
VEGF (DFS)	9	2.10 (1.26–3.49)	<0.01	71.44	0.25	0.02	2 left	1.77 (1.11–2.84)	
MVD (OS)	18	1.39 (1.22–1.58)	<0.01	83.14	0.97	<0.01	6 left	1.22 (1.07–1.40)	
Racial descent		1.44 (1.23–1.68)	<0.01	83.14	—	—	—	—	
America	2	1.57 (0.50–4.95)	0.44	73.92	—	—	—	—	0.55
Asia	7	1.35 (1.12–1.63)	<0.01	84.59	—	—	—	—	
Europe	9	1.62 (1.24–2.12)	<0.01	81.06	—	—	—	—	
Age		1.44 (1.1–1.89)	<0.01	82.78	—	—	—	—	
<65	6	2.17 (1.23–3.83)	<0.01	81.66	—	—	—	—	0.11
≥65	6	1.28 (0.94–1.74)	0.11	86.31	—	—	—	—	
Follow-up		1.41 (1.21–1.63)	<0.01	83.14	—	—	—	—	
<60 months	9	1.35 (1.14–1.60)	<0.01	82.99	—	—	—	—	0.33
≥60 months	9	1.60 (1.19–2.16)	<0.01	84.44	—	—	—	—	

DFS: disease-free survival; HR: hazard ratio; OS: overall survival; VEGF: vascular endothelial growth factor.

**Table 2:** Pooled ORs for the association between VEGF expression and clinical and pathology features.

	Number of studies	Pooled OR (95% CI)	*P* value
Gender^1^	13	1.08 (0.84–1.38)	0.55
Distant metastasis^2^	8	4.22 (2.93–6.06)	<0.01
Lymph node metastasis^2^	15	2.51 (1.51–4.15)	<0.01
Lymph metastasis^2^	7	1.57 (0.97–2.54)	0.06
Vascular metastasis^2^	9	2.38 (1.49–3.79)	<0.01
TNM^3^	13	1.65 (0.92–2.96)	0.09

OR: odds ratio; VEGF: vascular endothelial growth factor.

^1^Male versus female; ^2^metastasis of positive versus metastasis of negative; ^3^I and II versus III and IV.

**Table 3:** Pooled ORs for the association between MVD expression and clinical and pathology features both in qualitative and quantitative analyses.

	Qualitative analyses			Quantitative analyses				
	Number of studies	Pooled OR (95% CI)	*P* value	Number of studies	MD (95% CI)	*P* value	SMD (95% CI)	*P* value
Gender^1^	8	0.94 (0.71–1.24)	0.66	11	−4.43 (−18.82–9.96)	0.55	−0.76 (−1.36–0.15)	0.01
Grade^2^	11	1.47 (0.86–2.49)	0.15	—	—	—	—	—
Distant metastasis^3^	—	—	—	8	13.14 (−1.70–27.98)	0.08	1.43 (0.27–2.59)	0.02
Lymph node metastasis^3^	8	1.84 (1.19–2.85)	<0.01	10	7.97 (3.64–12.31)	<0.01	0.45 (0.22–0.69)	<0.01
Lymph metastasis^3^	5	1.76 (0.97–3.20)	0.06	6	7.15 (3.86–10.44)	<0.01	0.46 (0.28–0.64)	<0.01
Vascular metastasis^3^	7	1.43 (1.06–1.92)	0.02	8	13.99 (5.15–22.83)	<0.01	0.60 (0.26–0.95)	<0.01

MD: mean difference; MVD: microvessel density; OR: odds ratio; SMD: standardized mean difference.

^1^Male versus female, ^2^well and moderate versus poor and mucinous, and ^3^metastasis of positive versus metastasis of negative.
