# Supplementary material for: Can Vascular Endothelial Growth Factor and Microvessel Density Be Used as Prognostic Biomarkers for Colorectal Cancer? A Systematic Review and Meta-Analysis
Source: ScientificWorldJournal. 2014 Mar 27;2014:102736. doi: 10.1155/2014/102736 (PMC3985294; doi:10.1155/2014/102736)
Supplement: Supplementary file 1 — Online Resource 1 showed the quality assessment questionnaire, which was used to weight the quality and applicability of the included studies from research design, laboratory methodology and analysis of the results. Online Resources 2 summarized the main characteristics of the VEGF related studies, and the characteristics included detail information of the study population, median months of follow-up, HR value, adjusted variable in Cox analysis and quality scoring of each article. Online Resources 3 summarized the main characteristics of the MVD related studies, and the characteristics included detail information of the study population, median months of follow-up, HR value, adjusted variable in Cox analysis and quality scoring of each article. Online Resources 4 described the determination method of cut-off criteria and the specific cut-off value for VEGF/MVD in each study. And the cut-off value was used to assess the expression level of VEGF or MVD. [file 102736.f1.pdf]

Online Resource 1 Quality assessment questionnaire

1. Research Design

1) Definition of Study Objectives

a) Patient selection criteria, including histological type, disease stage, and primary or advanced: defined (1 point) or not defined (0 points).

b) Number of non-assessable samples with exclusion causes: defined (1 point) or not defined (0 points).

c) Whether patients had received chemotherapy or radiotherapy before surgery: defined (1 point) or not defined (0 points).

d) Description of treatment status after surgery: defined (1 point) or not defined (0 points).

2) Study design: prospective (2 points), retrospective (1 point), or not defined (0 points).

3) Statistical methods and test descriptions: defined (1 point) or not defined (0 points).

2. Laboratory Methodology

1) Observers blinded to the clinicopathological data: blind (1 point) or unblinded or not defined (0 points).

2) Test reproducibility control including 2 or more investigators testing the samples: defined (1 point) or not defined or fewer than 2 investigators (0 points).

3) Detailed description of the tests:

a) Experimental method: defined (1 point) or not defined (0 points).

b) The type of antibodies/antigens: defined (1 point) or not defined (0 points).

c) Coloration and reading methods: defined (1 point) or not defined (0 points).

4) Description of the negative or positive control procedures: defined (1 point) or not defined (0 points).

5) Definition of the level of positivity or negativity of the test (or cut-off value): defined (1 point) or not defined (0 points).

3. Analysis of the Results

1) The range or median of follow-up: both (2 points), either (1 point), or none (0 points).

2) Follow-up description: the percentage of loss to follow-up >20% or unknown (0 points), 5-20% (1 point), or <5% (2 points).

3) Survival analysis of the prognostic factors for survival: report of the multivariate analysis of hazard ratios and their confidence intervals (2 points), univariate analysis of the prognostic factors for survival or multivariate analysis results without evaluation of the hazard ratios and confidence intervals (1 point), or not reported or inadequate (0 points).

Online Resource 2 Main Characteristics of The Studies Including in The Analysis on Association Between VEGF and Survival of Colorectal Cancer Patients

Authors/ Year of publication	Country	Sample size (C/R)	TNM	No. of positive VEGF	Median months of follow-up	HR (95%CI)	Survival analysis	Quality score[1]	Adjusted variable in Cox analysis
Zhou et al 2011	China	141 (61/80)	M[2]	99	59	3.39(1.00-11.36)[3]	DFS	aab	diabetes, lymph nodes metastasis, COX-2,MMP-2, CEA
Kwon et al 2010	Korea	132	M	30	19.25	4.78(1.15-19.94)<br>1.28(0.41-4.03)	OS<br>DFS	bca	age, tumor size, tumor depth, IL6, CRP, CEA,
Liang et al 2010	China	114 (114/0)	M	56	53	0.73(0.54-0.99)<br>0.79(0.54-1.14)	OS<br>DFS	bab	differentiation, lymph nodal metastasis, lymphocytic infiltration, depth of invasion, distant metastasis, TNM, SPARC, MVD
Barresi et al 2010	Italy	52	L[4]	25	-	2.58(1.07-6.22)[3]	OS	bac	age, MVD
Hong et al 2009	Korea	31 (19/12)	A[5]	-	17.6[6]	1.66(0.48-5.77)<br>1.54(0.72-3.30)	OS<br>DFS	bab	CA9
Cao et al 2009	China	71	M	40	-	1.13(0.41-3.09)	OS	bac	HIF-1α
Toiyama Eet al 2009	Japan	40 (0/40)	M	-	41	30.25(1.72-529.68)	OS	bba	lymph node metastasis
Alabi et al 2009	UK	93 (53/40)	M	10	54	7.17(1.55-33.1)	DFS	bcb	TNM
Wei et al 2009	Taiwan	86 (57/29)	M	-	36	0.85(0.27-2.75)	OS	bba	age, sex, tumor location, TNM, CEA, PIGF, sFLT-1
Miyazaki et al 2008	Japan	127 (74/53)	M	63	44	1.91(1.06-3.50)<br>1.99(1.00-3.96)	OS<br>DFS	bcb	duck's stage
Zafirellis et al 2008	Greece	117 (78/39)	M	49	64	3.50(1.90-6.50)	DFS	aab	age, TNM
Giralt et al 2006	Spain	81 (0/81)	A	43	53	1.98(0.81-4.80)	DFS	aab	pathological stage

Ferroni et al 2005	Italy	69 (45/24)	M	27	76.3	5.15(1.10-24.10)	OS	aba	duck's stage
Boxer et al 2005	UK	56 (37/19)	L	12	79	4.23(1.12-15.98)[3]	OS	bbb	sex, age, duke's stage, location
Kojima et al 2005	Japan	65	M	24	-	2.31(0.61-8.78)	OS	bab	morphometric parameters of tumor, lymphatic invasion
Tamura et al 2004	Japan	49 (26/23)	M	21	102[6]	3.20(1.41-7.29)	OS	cab	No. of pulmonary metastases, duke's stage, CEA
Kaio et al 2003	Japan	152	M	40	61.2[6]	7.73(1.15-51.84)	OS	bab	lymph node metastasis, lymph and venous inavasion, PD-ECGF, MVD, se(a2)-si(ai), histological, differentiation
Khorana ett al 2003	USA	131 (131/0)	M	45	60	0.57(0.34-0.95)	OS	baa	age, adjuvant chemotherapy, AJCC's stage, EGFR
White et al 2002	UK	84 (62/22)	M	62	69.3	3.81(1.09-13.36)<br>4.13(1.18-14.46)	OS<br>DFS	bab	duck's stage, venous inavasion
Ishigami et al 1999	Japan	60 (31/29)	M	11	30.1	1.94(1.23-3.06)	OS	bcb	depth of tumour infiltration, maximum diameter, tumour differentiation

Footnotes: [1]Quality score present research design, lab method and statistical analysis respectively; [2]M means studies focus on patients of all stages(mixed); [3]Data extrapolated; [4]L means studies focus on limited forms (TNM I/II); [5]A means studies focus on advanced forms (TNM III/IV); [6]Mean months of follow-up.

Abbreviations: C, colon; CI, confidence interval; CA9, carbonic anhydrase 9; CEA, carcinoembryonic antigen; COX-2, cyclooxygenase-2; CRP, C-reactive protein; DFS, disease free survival; HIF-1α, hypoxia-inducible factor 1 alpha; EGFR, epidermal growth factor receptor; HR, hazard ratio; IL-6, interleukin-6; OS , overall survival; PD-ECGF, platelet-derived growth factor; PIGF, lacenta growth factor; R, rectum; sFLT-1, soluble secreted form of Flt-1; SPARC, secreted protein, acidic and rich in cysteine; VEGF, vascular endothelial growth factor.

Online Resource 3 Main Characteristics of The Studies Including in The Analysis on The Association Between MVD and Survival of Colorectal Cancer Patients

Authors Year of publication	Country	Study size (C/R)	TNM	No. of MVD positive	Median months of follow-up	HR(95%CI)	Survival analysis	Quality score[1]	Adjusted variable in Cox analysis
Moreira et al 2011	Brazil	60	M[2]	-	64.08	3.36(1.10-11.2)	OS	caa	TNM
Barresi et al 2010	Italy	52	L[3]	26	-	2.15(0.88-5.22)[5]	OS	bac	age, VEGF
Nanashima et al 2009	Japan	139 (75/64)	A[4]	-	52	2.71(1.15-6.42)	OS	baa	No. of tumor, node metastasis, CEA, classification, distribution of tumor,surgical margin, fibrous pseudocapsular formation, differentiation
Yodavudh et al 2008	Thailand	119 (72/47)	M	-	-	1.94(1.05-3.61)	OS	aab	differention, lymphatic vascular and perineural invasion, depth of invasion, distance metastasis, TNM
Rajaganeshan et al 2007	UK	109	A	39	33	1.10(1.03-1.18)	OS	baa	increasing size of the metastases, positive resection margin
Gulubova et al 2006	Bulgaria	106 (57/49)	M	54	32.2[6]	3.70(1.73-7.87)	OS	baa	age, histological,inflammatory infiltration, invasion in lymph vessels
Romani et al 2006	Italy	125	M	47	70.8	1.42(1.25, 1.60)	OS	bab	none
Chung et al 2006	China	101	A	-	36.3	1.02(0.97, 1.07)	OS	abb	angiopoietin-1,angiopoietin-2,lymphatic invasion, invasion of venous and perineural, pathological stage
Jubb et al 2006	USA	278	M	-	-	1.00(0.87-1.15)	OS	aaa	unknow
Boxer et al 2005	UK	56 (37/19)	L	27	79	0.31(0.08-1.15)	OS	bbb	dukes' stage, sex, age, location
Hasebe	Japan	157	L	-	62	3.40(1.50-7.50)[7]	OS	baa	fibroblast MIB-1 labeling index,

et al 2003			A			5.30(1.70-5.37)[7]	OS		dukes stage, vascular invasion
Miyagawa et al 2002	Netherlands	71	A	38	28	2.46(1.13-5.37)	OS	bab	differentiation, area of liver macrophage
Cianchi et al 2002	Italy	84	L	43	67	0.89(0.30-2.63)	OS	baa	age, gender, histology, differentiation, depth of invasion, invasive margin, lymphocytic infiltration, venous invasion
Vermeulen et al 1999	Belgium	116	M	59	30	3.26(1.41-7.55)	OS	aaa	age, tumor site, depth and size, histology, lymphatic invasion, lymph node metastasis, p53
Sternfeld et al 1999	German	146	M	61	-	2.31(1.33-4.00)	OS	bab	grade, angiosis, lymphangiosis, depth of penetration
Tanigawa et al 1997	Japan	133	M	66	72	3.39(1.80-6.40)	OS	baa	age, gender, tumor gross appearance, tumor site, tumor size, histology, tumor depth, lymphatic and vessel invasion, lymph node metastasis
Takebayashi et al 1996	Japan	163 (58/105)	M	-	92	1.04(0.98, 1.10)	OS	baa	dukes' stage, dThdPase

Footnotes: [1]Quality score present research design, lab method and statistical analysis respectively; [2]M means studies focus on patients of all stages(mixed); [3]L means studies focus on limited forms (TNM I/II); [4]A means studies focus on advanced forms (TNM III/IV); [5]Data extrapolated. [6]Mean months of follow-up; [7]Both data used in analysis.

Abbreviations: C, colon; CI confidence interval; DFS, disease free survival; dThdPase, thymidine phosphorylase; HR, hazard ratio ; MVD, microvessel density; OS , overall survival; R, rectum.

Online Resource 4 The Cut-Off Value of VEGF and MVD in Each Study

VEGF studies	Category	Cut-off value	MVD studies	Category	Cut-off value
Zhou et al 2011	-	-	Moreira et al 2011	Median	37
Kwon et al 2010	Arbitrary	825pg/ml	Barresi et al 2010	Median	28.25 vessel/mm^2
Liang et al 2010	Arbitrary	25%	Nanashima et al 2009	Median	238/mm^2
Barresi et al 2010	Arbitrary	Score>50	Yodavudh et al 2008	Median	20.5 vessel/HPF
Hong et al 2009	Arbitrary	Score>=80	Rajaganeshan et al 2007	-[1]	-
Cao et al 2009	Arbitrary	Score>=2	Gulubova et al 2006	Mean	58.64 vessel/mm^2
Toiyama et al 2009	-	-	Romani et al 2006	Arbitrary	21
Alabi et al 2009	Arbitrary	575pg/ml	Chung et al 2006	-	-
Wei et al 2009	Median	79.7pg/ml	Jubb et al 2006	-	-
Miyazaki et al 2008	Median	1840pg/ml	Boxer et al 2005	-	-
Zafirellis et al 2008	Arbitrary	75	Hasebe et al 2003	-	-
Giralt et al 2006	Arbitrary	Score>=4	Miyagawa et al 2002	Arbitrary	50
Ferroni et al 2005	Arbitrary	215pg/ml	Cianchi et al 2002	Median	45
Boxer et al 2005	Arbitrary	Score>=1	Vermeulen et al 1999	Median	75
Kojima et al 2005	Arbitrary	20%	Sternfeld et al 1999	Mean	75
Tamura et al 2004	Arbitrary	50%	Tanigawa et al 1997	Median	105
Kaio et al 2003	Arbitrary	10%	Takebayashi et al 1996	-	-
Khorana et al 2003	Arbitrary	2%			
White et al 2002	Arbitrary	Score>=2			
Ishigami et al 1999	Arbitrary	4.8[2]			

Footnotes: [1]no analysis; [2]VEGF T/N ratio.
